# Nrf2 Plays a Key Role in Erythropoiesis during Aging

**DOI:** 10.3390/antiox13040454

**Published:** 2024-04-12

**Authors:** Serge Cedrick Toya Mbiandjeu, Angela Siciliano, Alessandro Mattè, Enrica Federti, Massimiliano Perduca, Davide Melisi, Immacolata Andolfo, Angela Amoresano, Achille Iolascon, Maria Teresa Valenti, Francesco Turrini, Michele Bovi, Arianna Pisani, Antonio Recchiuti, Domenico Mattoscio, Veronica Riccardi, Luca Dalle Carbonare, Carlo Brugnara, Narla Mohandas, Lucia De Franceschi

**Affiliations:** 1 Department of Medicine, University of Verona, 37134 Verona, Italy; sergecedrick@gmail.com (S.C.T.M.); alessandro.matte@gmail.com (A.M.); davide.melisi@univr.it (D.M.); 2 Dipartimento Ingegneria per la Medicina di Innovazione—DIMI, University of Verona, 37134 Verona, Italy; angela.siciliano@univr.it (A.S.); federti.enrica@gmail.com (E.F.); veronica.riccardi@univr.it (V.R.); luca.dallecarbonare@univr.it (L.D.C.); 3 Department of Medicine, AOUI Verona, 37134 Verona, Italy; 4 Department of Biotechnology, University of Verona, 37134 Verona, Italy; massimiliano.perduca@univr.it (M.P.); michelebovi78@gmail.com (M.B.); ariannapisani92@gmail.com (A.P.); 5 Dipartimento di Medicina Molecolare e Biotecnologie Mediche, Università degli Studi di Napoli Federico II, 80131 Naples, Italy; immandolfo@gmail.com (I.A.); achille.iolascon@unina.it (A.I.); 6 CEINGE Biotecnologie Avanzate, 80131 Naples, Italy; 7 Department of Chimical Sciences, University Federico II, 80138 Naples, Italy; angela.amoresano@unina.it; 8 Department of Neuroscience, University of Verona, 37134 Verona, Italy; mariateresa.valenti@univr.it; 9 Department of Oncology, University of Torino, 10124 Torino, Italy; francesco.turrini@unito.it; 10 Department of Medical, Oral, and Biotechnology Science, “G. d’Annunzio” University Chieti–Pescara, 66013 Chieti, Italy; antonio.recchiuti@unich.it (A.R.); d.mattoscio@unich.it (D.M.); 11 Department of Laboratory Medicine, Boston Children’s Hospital, Boston, MA 02114, USA; carlo.brugnara@childrens.harvard.edu; 12 Department of Pathology, Harvard Medical School, Boston, MA 02114, USA; 13 New York Blood Center Enterprises, New York, NY 10065, USA; mnarla@nybc.org

**Keywords:** oxidation, astaxanthin, nanoparticles, PLGA, red cells, UPR system, autophagy, ATF6, GADD34, ineffective erythropoiesis

## Abstract

Aging is characterized by increased oxidation and reduced efficiency of cytoprotective mechanisms. Nuclear factor erythroid-2-related factor (Nrf2) is a key transcription factor, controlling the expression of multiple antioxidant proteins. Here, we show that Nrf2^−/−^ mice displayed an age-dependent anemia, due to the combined contributions of reduced red cell lifespan and ineffective erythropoiesis, suggesting a role of Nrf2 in erythroid biology during aging. Mechanistically, we found that the expression of antioxidants during aging is mediated by activation of Nrf2 function by peroxiredoxin-2. The absence of Nrf2 resulted in persistent oxidation and overactivation of adaptive systems such as the unfolded protein response (UPR) system and autophagy in Nrf2^−/−^ mouse erythroblasts. As Nrf2 is involved in the expression of autophagy-related proteins such as autophagy-related protein (Atg) 4-5 and p62, we found impairment of late phase of autophagy in Nrf2^−/−^ mouse erythroblasts. The overactivation of the UPR system and impaired autophagy drove apoptosis of Nrf2^−/−^ mouse erythroblasts via caspase-3 activation. As a proof of concept for the role of oxidation, we treated Nrf2^−/−^ mice with astaxanthin, an antioxidant, in the form of poly (lactic-co-glycolic acid) (PLGA)-loaded nanoparticles (ATS-NPs) to improve its bioavailability. ATS-NPs ameliorated the age-dependent anemia and decreased ineffective erythropoiesis in Nrf2^−/−^ mice. In summary, we propose that Nrf2 plays a key role in limiting age-related oxidation, ensuring erythroid maturation and growth during aging.

## 1. Introduction

Nuclear-factor erythroid derived 2 (Nrf2) is a redox-related transcription factor involved in acute phase response against oxidation [1,2]. The activation of Nrf2 results in the upregulation of genes carrying antioxidant-related element (ARE-) motif encoding for antioxidants and cytoprotectors such as catalase, peroxiredoxin-2 (Prdx2), NADPH dehydrogenase quinone-1 (Nqo1), heme-oxygenase-1 (Ho-1), Sestrin 2 (Srxn2) or thioredoxin (Trxn) [1,2,3,4,5,6,7,8]. Mice genetically lacking Nfr2 (Nrf2^−/−^) were noted to develop age-dependent immune-mediated anemia, which has been linked to the presence of naturally occurring antibody, promoting erythrophagocytosis [2,9]. However, mechanistic studies to further characterize the origin of the anemia in Nrf2^−/−^ mice are lacking. Recent studies in a mouse model for β-thalassemia demonstrate a persistent activation of Nrf2, which results in intracellular accumulation of inactive antioxidant systems [10,11]. This sustains cell engulfment and overactivation and impairment of autophagy, leading to apoptosis of erythroblasts and ineffective erythropoiesis. In murine β-thalassemia, we recently documented that functional collaboration between Nrf2 and Prdx2 regulates the extent of ineffective erythropoiesis [11]. Although progress has been made regarding Nrf2 function in response to oxidation, much remains to be defined regarding its role in normal and discorded erythropoiesis. 

Aging is a physiologic phenomenon characterized by increased oxidation and progressive decline in cell functions, with impairment of adaptive mechanisms such as the unfolded protein system (UPR) and autophagy [12,13]. Nrf2 is sensitive to overactivation of the UPR system and regulates the expression of autophagy-related proteins (Atgs) and key cargo proteins such as p62 [14,15]. Previous studies in various cell and animal models have shown that age-dependent dysregulation of these systems induces apoptosis by (i) cell engulfment with damaged proteins blocking autophagic flux and (ii) upregulation of UPR-system-related pro-apoptotic pathways such as the DNA damage protein inducing growth arrest (GADD34) and the caspase 3-dependent cascade [14]. In normal and stress erythropoiesis, increasing evidence indicates an important role of autophagy in supporting cell growth and differentiation [16,17,18]. However, limited insights are presently available regarding the contribution of the UPR system, except for the observation that Activating Transcription Factor 4 (ATF4) is involved in the cellular response to heme-mediated oxidation during erythroid maturation processes [17,18]. 

Here, we studied erythropoiesis in Nrf2^−/−^ mice. We found an age-dependent anemia due to decreased red cell lifespan in conjunction with ineffective erythropoiesis. We also documented the importance of Nrf2 activation and the upregulation of Prdx2 expression during erythropoiesis in old wild-type mice. Furthermore, in sorted Nrf2^−/−^ erythroid precursors, we documented a decline in cytoprotective mechanisms that included the UPR system and autophagy compared with wild-type animals, promoting ineffective erythropoiesis. As a proof of concept for defining the contribution of oxidation to ineffective erythropoiesis of Nrf2^−/−^ aged mice, we used lyophilized PLGA nanoparticles loaded with astaxanthin (ATS-NPs), a nontoxic and organic carotenoid with strong antioxidative properties [19] but without any pro-oxidative properties [20]. ATS-NP administration significantly decreased ineffective erythropoiesis and anemia in old Nrf2^−/−^ mice, mitigating endoplasmic reticulum (ER) stress and improving autophagy. Similar findings were noted with β-thalassemic mice treated with ATS-NPs. Collectively, our data indicate that Nrf2 is activated to support erythropoiesis during aging, mitigating age-related oxidation and modulating cytoprotective mechanisms such as the UPR system and autophagy.

## 2. Materials and Methods

### 2.1. Design of the Study

The present study was performed using C57BL/6J as control (wild-type; WT), Nrf2^−/−^ and Prdx2^−/−^ mouse strains at 4 and 12 months of age. We used female mice since erythropoietin responsiveness and iron homeostasis are affected by gender [21,22]. Mouse blood was collected by retro-orbital venipuncture in anesthetized mice using heparinized capillaries according to the general guidelines of the animal facility (CIRSAL) at the University of Verona. Where indicated, severe anemia was induced by intraperitoneal injection of doxorubicin (0.25 mg/kg body weight) [23]. Cells from the spleen and bone marrow were collected at day 9 following doxorubicin injection. Where indicated, mice were treated through intraperitoneal injection with astaxanthin-loaded PLGA nanoparticles (see also Section 2.6) at a dose of 2 mg/kg or vehicle every two days for four weeks. Hematological parameters, red cell indices and reticulocyte count were evaluated on an ADVIA 120 Hematology System (Siemens Healthcare GmbH, Henkestr, Germany) as previously described [23]. Hematocrit (Hct) and hemoglobin (Hb) were also manually determined. Red cells from wild-type or Nrf2^−/−^ mice were labeled with carboxyfluorescein succinimidyl ester dye (CFSE: 10 μM; Molecular probe, Invitrogen, Waltham, MA, USA) in PBS and 0.5% BSA for 20 min at 37 °C to assess red cell lifespan without any toxicity. CFSE covalently binds to intracellular and cell surface proteins with its succinidyl group [24,25]. Hemichromes bound to the red cell membrane and the extent of binding of naturally occurring anti-band 3 antibodies were assessed as previously reported [26]. 

### 2.2. Flow Cytometric Analysis of Erythropoiesis

Flow cytometric analysis of erythroid precursors from the bone marrow and spleen of mice was carried out using the CD44-Ter119 gating strategy as previously described [11]. Briefly, cells were centrifuged at 1500 rpm for 5 min at 4 °C and resuspended in BEPS (PBS 1X, BSA 1%, EDTA 2 mM, NaCl 25 mM). Cells were incubated first with CD16/32 (MA5-29707 eBioscience, San Diego, CA, USA) to block the Fc receptor for 15 min at 4 °C in the dark and then later incubated with CD45-APC-Cy7 (47-9459-42 Invitrogen, Waltham, MA, USA), CD44-FITC (SAB4700182 Sigma Aldrich, Merck Group, Darmstadt, Germany), CD71-PE and Ter119-APC (12-0711-82, A18447 eBioscience, San Diego, CA, USA) antibodies for 45 min at 4 °C in the dark. Cells were washed and centrifuged at 1500 rpm for 5 min at 4 °C and resuspended in BEPS, and 7AAD (A1310 Invitrogen, Waltham, MA, USA) was added immediately before the analysis to assess cell viability. Apoptotic erythroblasts were analyzed on CD44-Ter119-gated populations using the Annexin-V PE Apoptosis detection kit (88-8102-72 eBioscience, San Diego, CA, USA), following the manufacturer’s instructions [11]. ROS levels of the erythroid precursors were determined using the General Oxidative Stress Indicator, CM-H2DCFDA (C6827 LifeTechnologies, Carlsbad, CA, USA) on CD44-Ter119-gated populations as previously described [11].

Oxidative DNA damage of erythroid precursors was determined by 8OHdG flow cytometric analysis as previously described with some modifications. Briefly, erythroid precursors from mouse bone marrow were stained with CD44-FITC (SAB4700182 Sigma Aldrich, Merck Group, Darmstadt, Germany), CD71-PE and Ter119-BV480 (12-0711-82, 414-5921-82 eBiosciences, CA, USA) and fixed and permeabilized with BD Cytofix/Cytoperm and the BD Cytoperm Plus permeabilization reagent (554714 BD Biosciences, CA, USA), respectively. Cells were then stained with primary anti-8OHdG (sc-393871 Santa Cruz Biotechnology, Santa Cruz, CA, USA) and secondary anti-mouse eFluor647 (A-31571 Invitrogen, Waltham, MA, USA). All the analyses were performed with a FACS Canto-II^TM^ flow cytometer (Becton Dickinson, San Jose, CA, USA), and data were analyzed with the FlowJo v10 software (Tree Star, Ashland, OR, USA).

### 2.3. Analysis of Red Cells and Sorted Erythroid Precursors

Red cell membrane ghost and cytosol fractions were prepared as previously described [27]. For Prdx2 evaluation by Western blot, 100 mM of NEM was added to the lysis buffer to avoid possible artifacts related to Prdx2 oxidation during preparation. 

Erythroblasts (CD44^+^Ter119^+^FSC^high^) were sorted from WT and Nrf2^−/−^ mouse bone marrow using a FACS Aria-III^TM^ cell sorter (Becton Dickinson, San Jose, CA, USA) as previously reported [11]. Sorted cells were used for (i) immunoblot analysis; (ii) immunofluorescence assay; (iii) molecular analysis performed using QRT-PCR; (iv) CPP32/Caspase-3 Fluorometric protease assay (K105-25 BioVision, Milpitas, CA, USA; following manufacturer’s instructions); and (v) nuclear protein isolation, using the Q-proteome Nuclear Protein Kit (37582 Qiagen, Hilden, Germany) according to the manufacturer’s instructions). 

#### 2.3.1. Molecular Analysis of Erythroid Precursors

The protocols used for RNA isolation, cDNA preparation and qRT-PCR have been previously described [11]. Quantitative RT-PCR (qRT-PCR) was performed by the SYBR-green method, following standard protocols with an Applied Biosystems ABI PRISM 7900HT Sequence Detection system. Relative gene expression was calculated using the 2-DCt method, where DCt indicates the differences in the mean Ct between selected genes and the normalization control (Gapdh) [28]. The qRT-PCR primers for each gene were designed using Primer Express software version 2.0 (Life Technologies, Carlsbad, CA, USA). The primers used are reported in Table 1.

#### 2.3.2. Immunoblot Analysis of Red Cells and Erythroid Precursors

Red cells were separated into cytosol and membrane fractions as previously described [27]. Proteins from ghosts and the cytosol fraction were solubilized in reducing or non-reducing sample buffer (50 mM Tris, pH 6.8, 2% SDS, 10% glycerol, few grains of bromphenol blue added of 5% β-mercaptoethanol for reducing conditions) and analyzed by one-dimensional SDS–polyacrylamide gel electrophoresis. Gels were either stained with colloidal Coomassie or transferred to nitrocellulose membranes for immunoblot analysis with specific antibodies: anti-Prdx2 (ab109367 Clone1E8, Abcam, Cambridge, UK); anti-catalase (ab76110 Abcam, Cambridge, UK); anti-HSP70, anti-G6PD, anti-TrxR-1 and anti-Nqo1 (sc32239, sc373886, sc365658, sc271116 Santa Cruz Biotechnology, Texas, USA); anti-HSP90 (4874 Cell signaling Technology, Leiden, NL, USA); anti-Actin (SAB4301137 clone BIII-136; Sigma-Aldrich). Protein oxidation was detected by Oxyblot (S7150 Sigma-Aldrich), which allows the detection of carbonyl groups introduced into proteins by oxidative reactions according to the manufacturer’s instructions. 

Sorted erythroid precursors were prepared for immunoblot analysis as previously described [11]. The following specific antibodies were used: anti-NFkB-phospho-S536 (3033 Cell Signaling Technology, Leiden, NL, USA); anti-NFkB p65 and anti-Atg5 (8242, 12994 Cell Signaling Technology, Leiden, NL, USA); anti-Nrf2-phospho-S40 (ab76026 Clone EP1809Y, Abcam, Cambridge, UK); anti-Nrf2 (ab62352 Abcam, Cambridge, UK); anti Gadd34, anti-Lamp-1, anti-SQSTM1/P62, anti-Rab5 and anti-LC3A/B (ab9869, ab24170, ab109012, ab109534, ab62721 Abcam, Cambridge, UK); anti-APG7 (Atg7) (3615 ProSci, Poway, CA, USA); anti-ATF6 (70B1413.1 Novus Biologicals, Centennial, CO, USA); anti-CHOP (ma1-250 Thermo Fisher Scientific, Waltham, MA, USA); anti-Actin (SAB4301137 clone BIII-136; Sigma-Aldrich, Saint Louis, MO, USA) and anti-GAPDH (HPA040067 Sigma-Aldrich, Saint Louis, MO, USA) as loading controls. Blots were then developed using the Luminata Forte or Luminata Classico Western chemiluminescence reagents. Images were acquired using Image Quant Las Mini 4000 Digital Imaging System (GE Healthcare Life Sciences, Little Chalfont, UK), and densitometric analysis of band intensities was carried out using the ImageQuant TL 10.2 software (GE Healthcare Life Sciences).

### 2.4. Preparation and Characterization of Astaxanthin-Loaded PLGA Nanoparticles

A single-emulsion solvent evaporation method under sterile conditions at 20 °C was used for the synthesis of the astaxanthin (ATS)-loaded PLGA nanoparticles (NPs) [29]. Briefly, 20 mg of PLGA (7–17 kDa PLGA 50:50 with uncapped end groups (Sigma-Aldrich, St. Louis, MO, USA) and 1 mg of astaxanthin (CNLAB NUTRITION, ASIAN GROUP) were dissolved in a 2 mL mixture of 85% acetone and 15% ethanol. The organic phase was added dropwise to 20 mL of 0.5% aqueous polyvinyl alcohol surfactant under stirring. The obtained emulsion was maintained under stirring overnight to evaporate the solvents. The nanoparticles generated were collected by centrifugation at 13,000× *g* for 20 min at 10 °C and washed several times with 0.01 M phosphate-buffered saline, pH 7.4 (PBS), to remove residual solvents. Then, 5% mannitol was added as a cryoprotectant, and the NPs were divided into aliquots and lyophilized for storage. The mean size and zeta potential of NPs were estimated using the dynamic light scattering (DLS) technique (Nano ZetaSizer ZS, ZEN3600, Malvern Instruments, Malvern, Worcestershire, UK), and the synthesized NPs were suspended in PBS buffer (137 mM NaCl, 2.7 mM KCl, 10 mM Na_2_HPO_4_ and 1.8 mM KH_2_PO_4_) at the final concentration of 5 mg/mL; the samples were maintained at 25 °C. The encapsulation efficiency was calculated from an astaxanthin UV absorbance calibration curve prepared with different amounts of the carotenoid dissolved in dimethyl sulfoxide (DMSO). The calibration curve (Figure S6a) was set up by plotting the absorbance against the astaxanthin concentration. The calibration equation line was estimated using linear regression analysis, *y = ax + b*, with the correlation coefficient R^2^. Absorbance was recorded at 492 nm, the typical astaxanthin absorbance wavelength in this solvent, using empty nanoparticles’ absorbance as baseline correction.

To assess the stability of the nanoparticles and the ability to retain the entrapped astaxanthin over time both at physiological and DLS analysis temperatures (37 and 25 °C, respectively), a release study in PBS was performed. Samples were collected at different time intervals. Each ATS-NP aliquot was dissolved in DMSO, and the released carotenoid was spectrophotometrically quantified at 492 nm using the same calibration curve described above.

### 2.5. Mass Spectrometric Analysis of Plasma and Organ ATS-NP Distribution

The calibration curve for astaxanthin was developed for quantifying astaxanthin using the external standard method. The standard was dissolved in methanol acidified by 0.1% formic acid at a concentration of 1 mg/mL. Standard solutions of varying concentrations were prepared by serial dilution from the stock solution in a range from 0.1 ppb to 100 ppb. Details on extraction from serum and organs as well as the conditions for mass spectrometric analysis are described in the Supplementary Methods.

### 2.6. Splenic Macrophage Analysis and Erythrophagocytosis

Spleens were gently dissociated to generate single-cell suspensions with 70 µm nylon mesh strainers; fixed (30 min, rt) with 3% formalin; and incubated (15 min, 4 °C) with fluorochrome-labeled antibodies against CD45, F4/80 (a pan macrophage marker) and CD206 (an M2 macrophage marker). Cells were permeabilized (30 min, room temperature), counterstained with anti-Ter-119 antibody (15 min, 4 °C) and analyzed with a BD FACS Canto II flow cytometer for the simultaneous detection of RBCs phagocytosed by spleen macrophages expressing surface CD206. For microRNA (miRNA) expression analysis, spleens were homogenized in a lysis buffer with 10 mM β-mercaptoethanol, and the small RNA fraction was purified using Norgen isolation kits. miRNA (100 ng) was reverse transcribed with the miScript II kit (Qiagen), and 1 ng/reaction was used as a template for real-time PCR analysis. The relative expression of miRNAs was calculated using the 2^−ΔCt^ method [30], using SNORD95 as a housekeeping miRNA for the control of equal template loading. 

### 2.7. Statistics

A two-tailed unpaired Student *t*-test or two-way analysis of variance with Tukey’s multiple comparisons was used for data analyses. Wherever indicated, we used an unpaired Student *t*-test with Sidak correction or one-way ANOVA algorithm for repeated measures. Normality was assessed with the Shapiro–Wilk test. Data show values from individual mice and are presented as mean ± SD. Differences with *p* < 0.05 were considered significant.

## 3. Results

### 3.1. Nrf2^−/−^ Mice Show Accelerated Red Cell Senescence, Downregulation of Nrf2/ARE Antioxidant Systems and Age-Dependent Hyporegenerative Anemia

**Figure 1:**
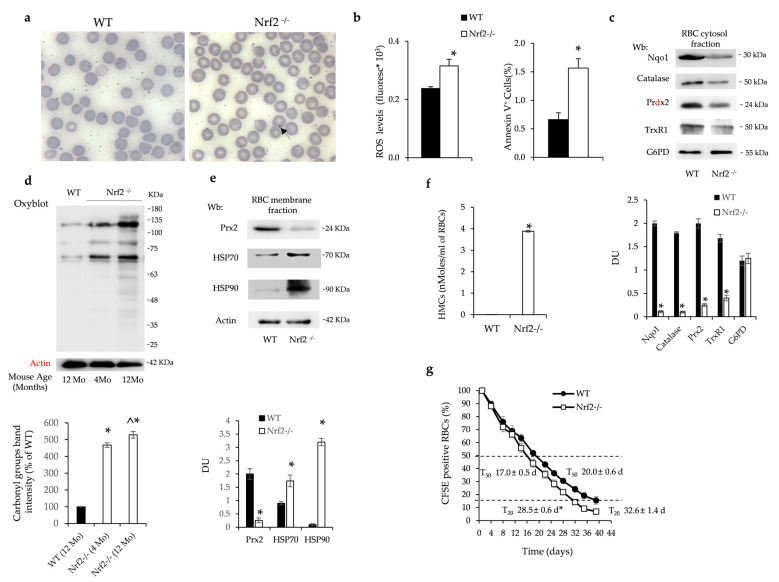
Nrf2^−/−^ mouse red cells display increased membrane oxidation resulting in accelerated senescence (**a**) Representative May–Grunwald–Giemsa staining for the morphology of red cells from wild-type (WT) and Nrf2^−/−^ mice. Arrow indicates Howell–Jolly body in Nrf2^−/−^ mouse red cells. Original magnification ×100. (**b**) ROS values in red cells and Annexin-V^+^ erythrocytes from WT and Nrf2^−/−^ mice. Data are presented as means ± SD (n = 4) * *p* < 0.05 compared to WT. (**c**) Western blot (Wb) analysis using specific antibodies against Nqo1, Catalase, Prdx2, TrxR1 and G6PD of the cytosolic fraction of red cells from WT and Nrf2^−/−^ mice. A representative gel of these four proteins is shown (upper panel). Densitometric analysis of immunoblots shown in the lower panel. Data are presented as means ± SD (n = 4); * *p* < 0.05 compared to WT mice. DU: densitometric unit. (**d**) Oxyblot analysis of the ghost fraction of red cells from WT and Nrf2^−/−^ mice at 4 and 12 months (Mo) of age. A representative gel of the other four is shown (upper panel). Actin is used as loading control. Densitometric analysis is shown in the lower panels. Data are presented as means ± SD (n = 4); * *p* < 0.05 compared to WT mice (12 Mo); ^ *p* < 0.05 compared to Nrf2^−/−^ mice at 4 Mo of age. (**e**) Wb analysis using specific antibodies against Prdx2, HSP70 and HSP90 of the ghost fraction of red cells from WT and Nrf2^−/−^ mice. A representative gel is shown (upper panel). Actin is used as protein loading control. Densitometric analysis of immunoblots is shown in the lower panel. Data are presented as means ± SD (n = 4); * *p* < 0.05 compared to WT mice. (**f**) Hemichrome quantification in red cells from WT and Nrf2^−/−^ mice at 12 months of age. Data are presented as means ± SD (n = 4); * *p* < 0.05 compared to WT mice. (**g**) Survival of CFSE-labeled red cells from WT and Nrf2^−/−^ mice at 12 months of age (n = 6–7). Data are mean ± SD. * *p* < 0.05 compared to T20 in WT mice.

Nrf2^−/−^ mice showed chronic mild normochromic normocytic anemia characterized by the presence of erythrocytes containing Howell–Jolly bodies (Table 2, Figure 1a). 

Nrf2^−/−^ mice developed age-dependent anemia characterized by a progressive increase in MCV and RDW with reduced reticulocyte count compared to either younger Nrf2^−/−^ mice or age-matched wild-type animals (Table 1). Nrf2^−/−^ mouse erythrocytes were characterized by increased production of reactive oxygen species (ROS) and higher amounts of Annexin-V+ red cells compared to wild-type animals (Figure 1b). The expression of Nrf2/ARE-related antioxidant and cytoprotective systems such as Nqo1, catalase, Prdx2 and thioredoxin reductase 1 (TrxR1) were significantly reduced in the cytoplasmic fraction of Nrf2^−/−^ mouse erythrocytes compared to wild-type red cells (Figure 1c). The expression of antioxidants was similarly reduced in young and old Nrf2^−/−^ mouse red cells (Figure S1a) and was associated with increased oxidation of red cell membrane proteins as determined by Oxyblot (Figure 1d). In agreement, in Nrf2^−/−^ mouse erythrocytes, we found (i) membrane translocation of classical heat shock protein-70 (HSP70) and -90 (HSP90) (Figure 1e) and (ii) reduced membrane translocation of Prdx2, which competes with hemichromes on the same band 3 binding sites (Figure 1e) [26]. We indeed found increased hemichromes bound to red cell membranes in Nrf2^−/−^ mice when compared to erythrocytes from wild-type animals (Figure 1f). The increased membrane oxidative damage was associated with increased binding of naturally occurring anti-band 3 antibodies (Figure S1b). The cumulative oxidative damage in Nrf2^−/−^ mouse red cells resulted in a decreased lifespan compared to that of wild-type erythrocytes (Figure 1g). No major difference in red cell survival was observed between young and old Nrf2^−/−^ mice (T50: 18.5 ± 0.5 days in 4-month-old Nrf2^−/−^ mice vs. 17.0 ± 0.5 days in 12-month-old Nrf2^−/−^ mice).

Taken together these data indicate that the absence of Nrf2 accelerates the senescence of circulating red cells, leading to the development of an age-dependent anemia. Furthermore, the progressive reduction in reticulocyte count implies perturbation of erythropoiesis during aging in mice genetically lacking Nrf2.

### 3.2. Nrf2^−/−^ Mice Develop Age-Dependent Ineffective Erythropoiesis Associated with Oxidative Damage Due to Lack of Nrf2/ARE-Dependent Antioxidant Systems

**Figure 2:**
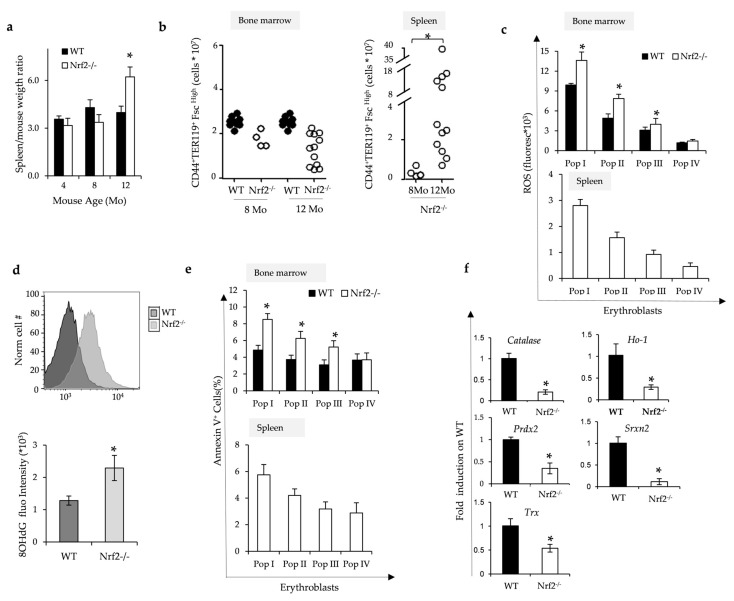
The absence of Nrf2 promotes ineffective erythropoiesis and increased oxidation of erythroblasts. (**a**) Spleen weight/mouse weight ratio of wild-type (WT) and Nrf2^−/−^ mice at 4, 8 and 12 months (Mo) of age. Data are presented as means ± SD (n = 4–12) * *p* < 0.05 compared to WT. (**b**) Flow cytometric analysis, combining CD44-Ter119 and cell size marker strategy (CD44+/Ter119+/FSC^high^), of the erythropoietic activity in the bone marrow and spleen from WT and Nrf2^−/−^ mice at 8 and 12 months (Mo) of age. Data are presented as single dots (n = 4–12) * *p* < 0.05 compared to WT. (**c**) ROS values in bone marrow and spleen erythroblasts from WT and Nrf2^−/−^ mice at 12 months of age. Data are presented as means ± SD, * *p* < 0.05 compared to WT. (**d**) DNA oxidative damage measured flow cytometrically as 8OHdG fluorescence intensity of erythroblast populations from WT and Nrf2^−/−^ mice at 12 months of age. Data are presented as means ± SD, * *p* < 0.05 compared to WT. (**e**) Amount of Annexin V+ cells in bone marrow and spleen erythroid precursors from Nrf2^−/−^ and WT mice as in (**c**); * *p* < 0.05 compared to WT. (**f**) mRNA expression of *catalase*, *Ho-1*, *Prdx2*, *Srxn2* and *Trx* by qRT-PCR in erythroblasts from WT and Nrf2^−/−^ mice (fold on WT). Experiments were performed in triplicate. * *p* < 0.01, Nrf2^−/−^ vs. WT mice. *p* value was calculated by *t*-test.

Nrf2^−/−^ mice displayed an age-dependent splenomegaly (Figure 2a) associated with extramedullary erythropoiesis due to increased splenic erythropoietic activity (Figure 2b). The erythroid maturation profile was similar in bone marrow from Nrf2^−/−^ mice when compared to wild-type animals (Figure S2a). However, we found (i) a marked increase in ROS values in maturating erythroblasts in the bone marrow of Nrf2^−/−^ mice from pop I, II and III corresponding to pro-, basophilic- and polychromatic erythroblasts, respectively (Figure 2c); (ii) increased amounts of 8-Hydroxydeoxyguanosine (8-OHdG) accumulation in total erythroblasts in Nrf2^−/−^ mice (Figure 2d); and (iii) an increase in Annexin-V positivity in erythroblasts from Nrf2^−/−^ mice compared to erythroblasts from wild-type animals (Figure 2e). As expected, sorted erythroblasts displayed reduced expression of Nrf2-dependent ARE genes such as *Catalase, Ho-1, Prdx2, Srxn1* and *Trxn*, with the finding confirmed by immunoblot analysis (Figure 2f and Figure S2b). Taken together, these results imply that the absence of Nrf2 increases oxidative stress and cell apoptosis, supporting an important role of Nrf2 in erythropoiesis during aging.

### 3.3. Nrf2 Is Activated during Erythropoiesis and Facilitated by Nuclear Translocation of Prdx2 during Aging

To assess whether Nrf2 may be important during erythropoiesis in aging mice, we evaluated Nrf2’s localization and activity in sorted erythroblasts from wild-type mice at 4 and 12 months of age. 

**Figure 3:**
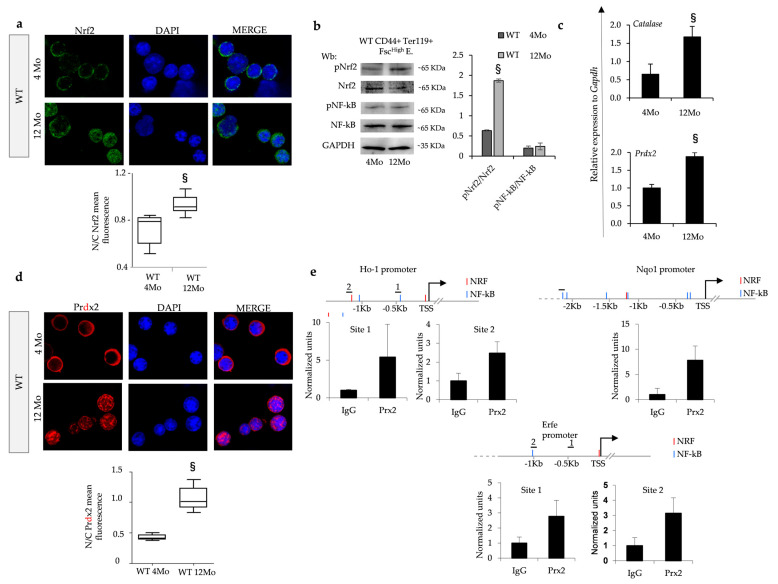
In aging wild-type mice, erythroblasts show age-dependent activation of Nrf2 associated with nuclear translocation of Prdx2, which is recruited in promoter regions of Nrf2. (**a**) Nrf2 immunostaining of sorted erythroid precursors from bone marrow of 4- and 12-month-old wild-type (WT) mice. DAPI was used to stain nuclei. Nrf2 mean fluorescence in the nucleus and cytoplasm was measured using ImageJ (https://imagej.net/ij/download.html). At least 25 cells were analyzed in 6 different fields of acquisition. Data are presented as median and minimum/maximum, with boxes indicating 25th–75th percentiles. § *p* < 0.05 compared to 4-month-old WT mice. Original magnification 100×. (**b**) Wb analysis with specific anti-phospho-Nrf2 (p-Nrf2), Nrf2, phospho-NF-kB(p-NF-kB) and NF-kB in sorted erythroid precursors in bone marrow of 4- and 12-month-old WT mice. GAPDH was used as protein loading control. A representative gel is shown. Densitometric analysis of immunoblots is shown in the right panel. Data are presented as means ± SD (n = 4); § *p* < 0.05 compared to 4-month-old WT mice. DU: densitometric unit (**c**) mRNA expression of *catalase* and *Prdx2* by qRT-PCR of erythroblasts from WT mice at 4 and 12 months of age. Data are mean ± SD (n = 6–8). Experiments were performed in triplicate. § *p* < 0.01, WT 4Mo vs. WT 12Mo mice. *p* value was calculated by *t*-test. (**d**) Prdx2 immunostaining of sorted erythroid precursors from bone marrow of 4- and 12-month-old wild-type (WT) mice. DAPI was used to stain nuclei. Prdx2 mean fluorescence in the nucleus and cytoplasm was measured using ImageJ. At least 40 cells were analyzed in 5 different fields of data acquisition. Data are presented as median and minimum/maximum, with boxes indicating 25th–75th percentiles; § *p* < 0.01, WT 4Mo vs. WT 12Mo mice. Original magnification 100×. (**e**) Predicted NRF and NF-kB binding sites in the indicated promoters are shown in red and blue, respectively. Horizontal bars indicate the regions amplified in the ChIP experiment. Histograms show qPCR quantification of a ChIP assay with the indicated antibodies from cells treated as above. Error bars indicate standard deviations.

As shown in Figure 3a, Nfr2 nuclear localization increased in sorted erythroid precursors from 12-month-old wild-type mice compared to younger 4-month animals (see also Figure S3a for brightfield images) in conjunction with an increase in the phospho-Nrf2 form in sorted erythroblasts from 12-month-old wild-type mice compared to younger animals (Figure 3b). No major change in the activation of NF-kB was evident in erythroid precursors from aging wild-type mice (Figure 3b). Indeed, we noted an age-dependent upregulation of Nrf2-related ARE genes such as catalase and Prdx2 in old wild-type animals compared to younger animals (Figure 3c). Since we previously described a functional collaboration between Nrf2 and Prdx2 supporting stress erythropoiesis [11], we evaluated Prdx2 distribution in erythroblasts from aging wild-type mice. This is important since in other cell models, Prdx2 has been shown to translocate to the nucleus to protect cells against oxidation-induced DNA damage [31,32,33]. We indeed noted nuclear translocation of Prdx2 in sorted erythroblasts from old wild-type mice compared to younger animals (Figure 3d and Figure S3b). This finding was further validated by immunoblot analysis of nuclear fraction from aged wild-type mice compared to the nuclear fraction from age-matched sorted erythroblasts from mice genetically lacking Prdx2 (Prdx2^−/−^ mice) (Figure S3c). 

To define the potential differences in the binding of Prdx2 to regulatory regions of the chromatin in old wild-type mouse erythroblasts, we performed a chromatin immunoprecipitation (ChIP) assay using a specific anti-Prdx2 antibody. We analyzed the putative promoter regions of the erythroferrone (*Erfe*), *Ho-1* and *Nqo1* genes to identify Nrf2 and NF-kB binding sites by MatInspector (www.genomatix.de) and looked for enrichment of immunoprecipitated chromatin using the anti-Prdx2 antibody as compared to control IgG (Figure 3e). We could indeed demonstrate the recruitment of Prdx2 to the promoter regions of *Erfe, Ho-1* and *Nqo1*, linking Nrf2 to *Erfe* gene expression for the first time. Importantly, we observed downregulation of *Erfe* in sorted erythroblasts from Nrf2^−/−^ mice (Figure S3d). 

Collectively, these findings indicate that Nrf2 activation during aging in wild-type mice involves Prdx2 nuclear translocation and is key for protecting cells against oxidation-induced DNA damage. 

### 3.4. Nrf2^−/−^ Mice Display Increased Sensitivity to Doxorubicin-Induced Stress Erythropoiesis 

Doxorubicin (Doxo) promotes severe oxidative stress and induces DNA damage, driving cells into senescence [23,34]. We evaluated the effect of Doxo treatment on erythropoiesis in Nrf2^−/−^ mice compared to wild-type mice. Following administration of Doxo for 9 days, we noted a significant reduction in Hct in Nrf2^−/−^ mice compared to wild-type mice (Figure S4a, upper panel) in association with a lower reticulocyte count (Figure S4a, lower panel). In agreement, we found a significant reduction in the total numbers of erythroblasts in both bone marrow and spleen in Doxo-treated Nrf2^−/−^ mice compared to wild-type mice (Figure S4b). Furthermore, Annexin V^+^ cells were significantly increased in polychromatic and orthochromatic erythroblasts from Nrf2^−/−^ mice as compared to wild-type animals (Figure S4c). Collectively, our findings indicate that mice genetically lacking Nrf2 are characterized by a blunted response to stress erythropoiesis induced by Doxo, by accelerating cell senescence and apoptosis. 

### 3.5. The Absence of Nrf2 Promotes Overactivation of UPR System and Impairment of Autophagy and Facilitates the Activation of Caspase-3 Pro-Apoptotic Pathway

**Figure 4:**
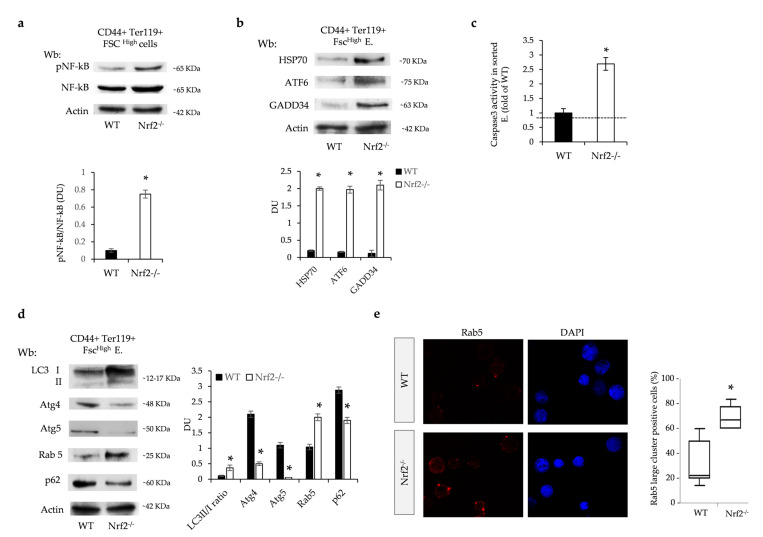
Sorted Nrf2^−/−^ mouse erythroblasts display overactivation of the system and impaired autophagy with caspase-3 pro-apoptotic pathway activation. (**a**) Wb analysis with specific anti-phospho-NF-kB (p-NF-kB) and NF-kB in sorted erythroid precursors from bone marrow of 12-month-old wild-type (WT) and Nrf2^−/−^ mice. Actin was used as protein loading control. One representative gel of the other four is shown (upper panel). Densitometric analysis of immunoblots is shown in the lower panel. Data are presented as means ± SD (n = 4); * *p* < 0.05 compared to WT mice. (**b**) Wb analysis with specific anti-HSP70, ATF6 and GADD34 in sorted erythroid precursors as in a. Densitometric analysis of immunoblots is shown in the lower panel. Data are presented as means ± SD (n = 4); * *p* < 0.05 compared to WT mice. (**c**) Caspase 3 activity determined by cleavage of a fluorescent substrate in sorted erythroid precursors from bone marrow of WT and Nrf2^−/−^ mice. Data are presented as means ± SD * *p* < 0.05 compared to WT. (**d**) Wb analysis with specific anti-LC3 I/II, Atg4, Atg5, Rab5 and p62 in sorted erythroid precursors from bone marrow of 12-month-old WT and Nrf2^−/−^ mice. Actin was used as protein loading control. Densitometric analysis of immunoblots is shown in the right panel. Data are presented as means ± SD (n = 4); * *p* < 0.05 compared to WT mice. (**e**) Rab5 immunostaining of sorted erythroid precursors from bone marrow of 12-month-old WT and Nrf2^−/−^ mice. DAPI was used to stain nuclei. Large clusters of positive cells were measured using ImageJ. At least 40 cells were analyzed in 8 different fields of acquisition. Data are presented as median and minimum/maximum, with boxes indicating 25–75th percentiles; * *p* < 0.05 compared to WT mice.

Since the absence of Nrf2 negatively impacts erythropoiesis during aging, we hypothesized that stress-sensitive transcription factor NF-kB might be activated to support stress erythropoiesis in Nrf2^−/−^ mice. Indeed, we found increased NF-kB activation in sorted erythroblasts from Nrf2^−/−^ mice compared to wild-type animals (Figure 4a). Other adaptive mechanisms against sustained oxidation such as UPR and autophagy could also be involved. In sorted Nrf2^−/−^ erythroid precursors, we found increased expression of (i) HSP70 and (ii) ATF6, which is one of the three UPR sensors and upregulates the expression of the pro-apoptotic protein GADD34 towards the caspase-3 cascade (Figure 4b). Indeed, we found a significantly increased activity of caspase-3 in sorted Nrf2^−/−^ mouse erythroblasts compared to wild-type cells (Figure 4c). 

Previous studies have shown that in the presence of severe or prolonged stress, the UPR system induces autophagy [35]. Given that efficient autophagy is required for erythroid maturation, we evaluated the expression of several key autophagy proteins in sorted erythroid precursors from the bone marrow of Nrf2^−/−^ and wild-type mice. We focused our analysis on (i) LC3 as an initiator of autophagy; (ii) Atg4, involved in the fusion of autophagosomes with lysosomes; (iii) Atg5, involved in the maturation of autophagosomes together with Rab5, which contributes to recycling endosomes and thereby regulates the fusion process between endosomes; and (iv) p62 as a cargo protein [36]. As shown in Figure 4d, we found increased LC3II conversion, associated with a reduction in Atg4, and Atg5 expression and accumulation of Rab5, suggesting a possible impairment of the terminal phases of autophagy in Nrf2^−/−^ erythroblasts (Figure 4d). Indeed, Nrf2^−/−^ erythroblasts displayed *punctae* of Rab5 organized in large clusters more abundantly than in wild-type erythroblasts (Figure 4e and Figure S5a). We further documented a reduced level of mRNA and protein p62 expression in Nrf2^−/−^ erythroblasts compared to wild-type cells (Figure 4e and Figure S5b). 

Thus, the absence of Nrf2 promotes premature cell senescence via impaired autophagy and overactivation of the UPR systems mainly involving ATF6-GADD34 towards the pro-apoptotic caspase-3 pathway. 

### 3.6. In Nrf2^−/−^ Mice, Treatment with Astaxanthin PLGA Nanoparticles Enhances Erythropoiesis and Improves Anemia

**Figure 5:**
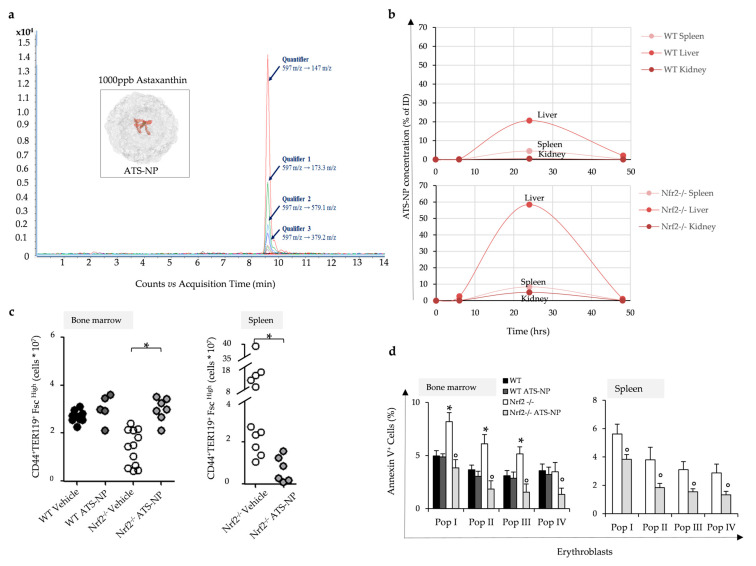
Astaxanthin PLGA nanoparticles improve Nrf2^−/−^ mouse ineffective erythropoiesis. (**a**) Extracted ion chromatogram for astaxanthin illustrates three qualifier transitions and was used to confirm molecule identification. Inset: ATS-NP: pictorial representation of PLGA nanoparticles embedding astaxanthin; the relationship between the different molecules is illustrative. (**b**) Organ distribution of astaxanthin molecules in the two mice strains during treatment determined by mass spectrometric analysis (see also Figure S6d and Table S1). (**c**) Flow cytometric analysis, combining CD44-Ter119 and cell size marker strategy (CD44+/Ter119+/FSC^high^), of the erythropoietic activity in the bone marrow and spleen from WT and Nrf2^−/−^ mice treated with vehicle or ATS-NPs (2 mg/kg every two days for four weeks). Data are presented as single dots (n = 5–12) * *p* < 0.05 compared to vehicle-treated WT mice. (**d**) Amount of Annexin V+ cells in erythroid precursors from bone marrow and spleen of WT and Nrf2^−/−^ mice treated as in (**c**). Data are presented as means ± SD; * *p* < 0.05 compared to WT mice, ° *p* < 0.05 compared to vehicle-treated mice.

Reduction in oxidative stress may beneficially impact Nrf2^−/−^ mouse erythropoiesis. Thus, we treated Nrf2^−/−^ mice with lyophilized PLGA nanoparticles loaded with astaxanthin (Figure 5a, see also stability of ATS-NP release at 25 °C and 37 °C in Supplementary Figure S6a–c). Astaxanthin (3,3′-dihydroxy-β-carotene-4,4′-dione) is a nontoxic and organic carotenoid with potent antioxidative activity with no pro-oxidative properties. Its unique molecular structure quenches singlet oxygen and scavenges free radicals, preventing lipid peroxidation [37]. Previous studies have shown that astaxanthin has a wide variety of biological effects including anti-inflammatory, antiapoptotic, neuroprotective and cardioprotective effects [37,38,39,40,41]. 

The organ distribution of ATS was determined by mass spectrometric analysis at 24 h after ATS-NP administration (Figure 5a,b, see also Figure S6d). ATS was identified in the spleen, liver and kidney in both mouse strains (Figure 5a,b). Higher levels of ATS were found in Nrf2^−/−^ mouse organs when compared to wild-type animals (Figure 5b). Previous studies have shown that PLGA-NPs are generally cleared by phagocytic uptake by (i) the spleen and liver, (ii) hepatic filtration and (iii) kidney extraction [42]. 

ATS-NP treatment decreased the extent of anemia of Nrf2^−/−^ mice by reducing ineffective erythropoiesis and improving the quality control process for red cell production. As shown in Figure 5c, ATS-NPs increase bone marrow erythropoietic activity and decrease extramedullary erythropoiesis (Figure S7a). This was associated with a significant reduction in apoptosis of erythroblasts from ATS-NP-treated Nrf2^−/−^ mice compared to vehicle-treated animals (Figure 5d). 

**Figure 6:**
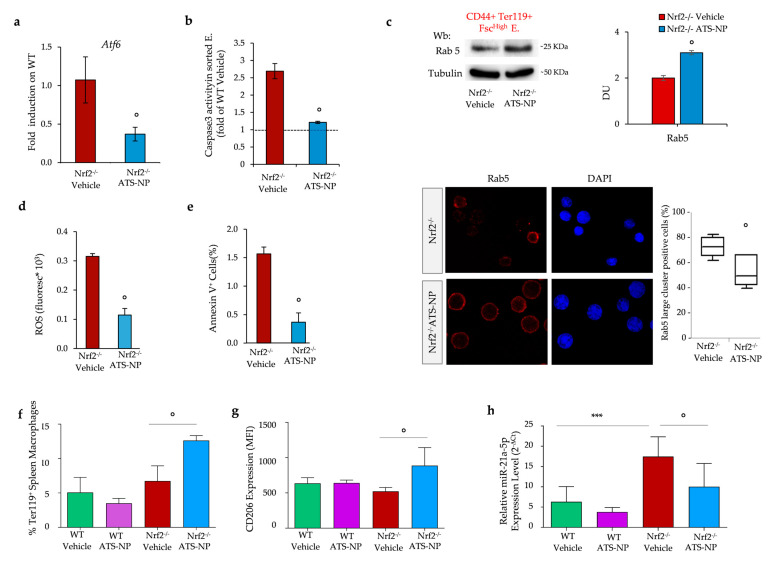
Astaxanthin PLGA nanoparticles reduce oxidation, prevent overactivation of adaptative systems in erythroblasts and modulate splenic macrophages towards a pro-resolving pattern in Nrf2^−/−^ mice. (**a**) mRNA expression of *Atf6* by qRT-PCR on the erythroblasts from vehicle and ATS-NP treated Nrf2^−/−^ mice. Data are mean ± SD (n = 6–8). Experiments were performed in triplicate. ° *p* < 0.01, Nrf2^−/−^ vehicle vs. Nrf2^−/−^ ATS-NP mice by *t*-test; (**b**) Caspase 3 activity determined by its cleavage of a fluorescent substrate in sorted erythroid precursors from bone marrow of Nrf2^−/−^ mice treated as in (**a**); ° *p* < 0.01, Nrf2^−/−^ vehicle vs. Nrf2^−/−^ ATS-NP mice by *t*-test. (**c**) Upper panel: Wb analysis with specific anti-Rab5 in sorted erythroid precursors from bone marrow of Nrf2^−/−^ mice treated as in (**a**). Densitometric analysis of immunoblots is shown in the right panel. Data are presented as means ± SD (n = 4); ° *p* < 0.01, Nrf2^−/−^ vehicle vs. Nrf2^−/−^ ATS-NP mice by *t*-test. Lower panel: Rab5 immunostaining of sorted erythroid precursors from bone marrow of Nrf2^−/−^ mice treated as in (**a**). DAPI was used to stain nuclei. Large clusters of positive cells were measured using ImageJ. At least 35 cells were analyzed in 5 different fields of acquisition. Data are presented as median and minimum/maximum, with boxes indicating 25th–75th percentiles. (**d**) ROS levels in red cells and (**e**) Annexin-V^+^ erythrocytes from Nrf2^−/−^ mice treated as in (**a**). Data are presented as mean ± SD (n = 4) ° *p* < 0.05 compared to vehicle-treated mice. (**f**) Flow cytometry analysis of phagocytosis of Ter-119^+^ cells, membrane expression of CD206 (**g**) and real-time PCR analysis of mir-21-5p (**h**) in splenic macrophages. Data are mean ± SD (n = 6); ***, *p* < 0.001; ° *p* < 0.05 by 1-way ANOVA.

The beneficial effects of ATS-NPs on Nrf2^−/−^ mouse erythropoiesis were also supported by (i) the downregulation of *Atf6* expression (Figure 6a), (ii) a reduction in caspase-3 activity (Figure 6b) and (iii) the improvement of autophagy with the clearance of large Rab5 clusters (Figure 6c lower panel).

The improvement of the quality control process during erythroid maturation promoted by ATS-NP treatment resulted in the amelioration of anemia and red cell features in Nrf2^−/−^ mice (Table 3). 

In ATS-NP-treated Nrf2^−/−^ mice, we also found decreased (i) ROS, (ii) AnnexinV+ for red cells, (iii) red cell membrane protein oxidation and (iv) red cell membrane translocation of HSP70 and HSP90 (Figure 6d,e and Figure S7b,c). This was associated with the optimization of erythrophagocytosis by Nrf2^−/−^ mouse macrophages (Figure 6f) and a reduction in apoptotic Ly6G^+^ neutrophils (Figure S7d). Furthermore, in Nrf2^−/−^ mice, ATS-NP treatment enhanced the expression of CD206, a classical surface marker of M2 polarization, and reduced the relative expression of miR-21-5p, a microRNA that promotes the acquisition of an M1 inflammatory macrophage phenotype [43], which was significantly over-expressed in Nrf2^−/−^ mice compared to wild-type mice (Figure 6g,h). Taken together, our data indicate that ATS-NPs support stress erythropoiesis, reduce erythrophagocytosis and optimize spleen macrophage function skewing toward a pro-resolving, M2 phenotype in cells lacking Nrf2. 

## 4. Discussion

In the present study, we show the key role of Nrf2 in supporting erythropoiesis and the quality control processes involved in erythroid maturation during mouse aging. We confirm that the absence of Nrf2 results in the impairment of red cell antioxidant machinery [1,2,9], promoting an accelerated removal of Nrf2^−/−^ mouse erythrocytes. This was associated with an age-dependent anemia characterized by reduced reticulocyte count, suggesting a perturbation of erythropoiesis. The presence of ineffective erythropoiesis in 12-month-old Nrf2^−/−^ mice was documented by (i) extramedullary erythropoiesis, (ii) increased ROS throughout erythroblast maturation, (iii) oxidative DNA damage in erythroblasts and (iv) increased cell apoptosis at all stages of erythroblast maturation. We also demonstrated that Nrf2 is activated in wild-type erythropoiesis during mouse aging, with slight but stable activation of NF-kB in sorted erythroblasts from young vs. old wild-type mice. This agrees with previous observations of primarily cytoplasmic localization of Nrf2 in maturating erythroid cells from healthy adults [44]. The activation of NF-kB is a possible compensatory mechanism for the absence of Nrf2 in erythroblasts from old Nrf2^−/−^ mice when compared to younger animals, as suggested by other models [45,46]. A similar observation has been also reported in models of anemia in the terminal stage of renal disease [47,48]. Indoxyl sulfate, a uremic toxin, accelerates cell senescence towards apoptosis and reduces EPO responsiveness in both K562- and CD34+-derived cells by the inhibition of Nrf2 and modulation of NF-kB activity [47,48]. The importance of Nrf2 in cell growth and maturation of erythroblasts during aging is further supported by (i) the upregulation of Prdx2; (ii) Prdx2 nuclear translocation; and (iii) Prdx2 recruitment on the promoter region of *Erfe*, *Ho-1* and *Nqo1*, linking Nrf2 to *Erfe* gene expression in erythroblasts from wild-type mice for the first time, in addition to the reported role of the signal transducer and activator of transcription-5 (STAT5) in *Erfe* gene expression [49,50]. Collectively, these data indicate the importance of Nrf2 in erythropoiesis during aging and the collaborative action of Nrf2 and Prdx2 in limiting oxidation and assisting in cell growth and maturation in erythroblasts. The blunted response to stress erythropoiesis induced by Doxo, which promotes oxidation DNA damage and accelerates the aging phenomenon, further supports the increased susceptibility of Nrf2^−/−^ mice to oxidative stress. 

Perturbations of cellular adaptative mechanisms against oxidation such as the unfolded protein response (UPR) system and autophagy have been recently described in pathologic erythropoiesis such as β-thalassemia [16,18,51]. In old Nrf2^−/−^ mice, prolonged oxidation resulted in the sustained activation of the UPR systems, mainly involving ATF6, promoting the upregulation of HSP70 and GADD34 as part of the EIF2a-ATF4 signaling pathway towards autophagy [16,18,52]. However, in erythroblasts from Nrf2^−/−^ mice, we observed an impairment of late-phase autophagy. This might be favored by the downregulation of Nrf2-dependent Sestrin-2 expression, which contributes to the modulation of autophagy [53]. In addition, we note that Atg4, 5 and p62 genes also contain the ARE-sequence, modulated by Nrf2 [54]. Thus, we were not surprised that in sorted Nrf2^−/−^ erythroblasts, Atg4, 5 and p62 expression was downregulated compared to wild-type cells [20,55,56,57,58]. This resulted in the accumulation of Rab5 organized in large clusters, supporting the impairment of autophagy in Nrf2^−/−^ erythroblasts. The cumulative effect of sustained oxidation, prolonged overactivation of the UPR system and impairment of autophagy triggers the caspase-3 pro-apoptotic system, contributing to the ineffective erythropoiesis in old Nrf2^−/−^ mice. 

As a proof of concept, our experiments with novel ATS-NPs support this working model. We found that in Nrf2^−/−^ mice, ATS-NPs ameliorate age-dependent macrocytic anemia and improve ineffective erythropoiesis. The reduction in intracellular oxidation by ATS-NPs rebalances the overwhelmed autophagy observed in Nrf2^−/−^ mouse erythroblasts. This results in a significant reduction in the accumulation of large Rab5 aggregates in erythroblasts from ATS-NP-treated Nrf2^−/−^ mice. Of note, we observed a difference in the amounts of ATS detected in the liver and spleen from Nrf2^−/−^ vs. wild-type mice, which might be related to pro-inflammatory vs. pro-resolving profile of macrophages in the spleen and liver lacking the expression of Nrf2 as previously noted in other disease models [57,58]. Indeed, ATS-NPs reprogram splenic macrophage from a pro-inflammatory to a pro-resolving M2 profile [43]. 

**Figure 7:**
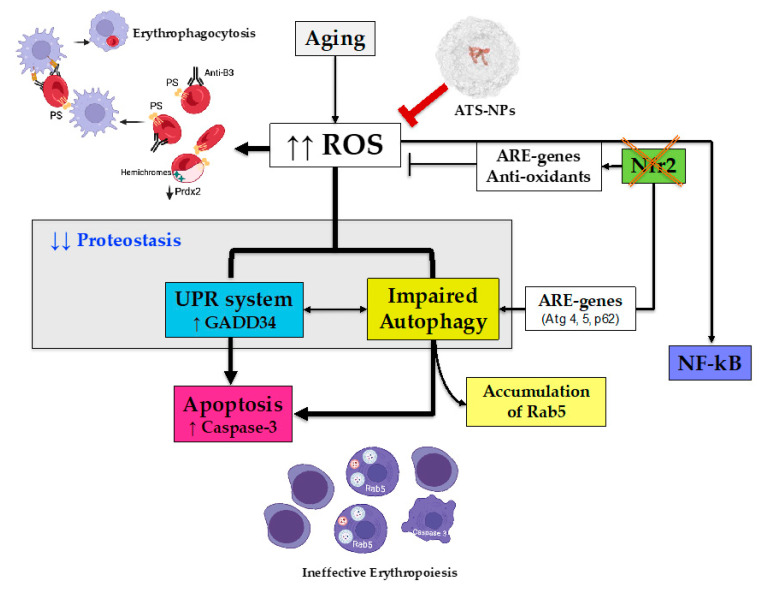
Schematic diagram of the role of Nrf2 in erythropoiesis during aging and the protective effects of astaxanthin PLGA nanoparticles. Aging is associated with increased ROS production, which is limited by the activation of Nrf2. This results in the upregulation of ARE genes encoding for antioxidants and cytoprotective systems as well as by the activation of adaptative mechanisms such as the UPR system to face ER stress and autophagy to clear damaged proteins. The absence of Nrf2 (Nrf2^−/−^ mice) negatively affects the antioxidant cell machinery, resulting in severe and sustained oxidation. Nrf2^−/−^ mouse red cells display severe membrane oxidation, exposition of phosphatidylserine, membrane binding of hemichromes and reduced expression of antioxidants and cytoprotective systems such as Prdx2. Red cell membrane protein oxidation favors band 3 protein clusterization, which is recognized by the naturally occurring anti-band 3 IgG antibodies. Both mechanisms drive Nrf2^−/−^ mouse red cells towards erythrophagocytosis by splenic macrophages. In erythroblasts lacking Nrf2, the prolonged and severe oxidation due to the downregulation of antioxidants and cytoprotective systems promotes intense ER stress with overactivation of the UPR system and autophagy. Although the persistence of oxidative stress promotes compensatory activation of NF-kB, this is insufficient to prevent the overwhelming of proteostasis with impairment autophagy and accumulation of Rab5. This drives Nrf2^−/−^ erythroblasts towards apoptosis via the caspase-3 pathway, resulting in ineffective erythropoiesis. ATS-NPs act as efficient antioxidants preventing the deleterious effects of the absence of Nrf2 on erythropoiesis and red cells during aging. PS: phosphatidylserine; ER: endoplasmic reticulum; UPR: unfolded protein response; ARE-: antioxidant-related element; ROS: reactive oxygen species; Prdx2: peroxiredoxin-2; Atg: autophagy-related protein; GADD34: growth arrest and DNA damage-inducible protein 34; PLGA: poly(lactic-co-glycolic acid).

In conclusion, we propose that Nrf2 is a key transcriptional factor in erythropoiesis protecting against oxidative damage during aging (Figure 7). In aging mice, the absence of Nrf2 results in severe oxidative damage and overactivation of the UPR and autophagy resulting in perturbation of proteostasis, leading to apoptosis of erythroblasts. The senostatic effect of ATS-NPs decreases anemia and ineffective erythropoiesis in Nrf2^−/−^ mice, preventing the overwhelming of adaptative systems and ensuring erythroid survival and maturation. 

## Figures and Tables

**Table 1:** Primers.

Gene	Forward Primer Sequence (5’→ 3’)	Reverse Primer Sequence (5’→ 3’)
*Gapdh*	CCACATCGCTCAGACACCAT	AGTTAAAAGCAGCCCTGGTGAC
*Erfe* *Atf6* *P62* *Txn* *Srxn2* *Prdx2* *Ho-1*	ATGGGGCTGGAGAACAGC GCCCGGTGAATGGAAAACTT AGCGGTTACTCACTCCATGGAAGA CATGGCCAACAAAATCATGCAGCT TTTGGCAGAGACAT CGCCTAGTCCAGGCCTTTCCAA GCACAGGGTGACAGAAGAG	TGGCATTGTCCAAGAAGACA GTCTCCTCAGCACAGCGATA CTTGGGGAGGTTTCGTCTCT CGATCTGTTCAATTTTCGTTGGG ACTTTCAGCGTGGCTGGG GATGGTGTCACTGCCGGG GTCAGCATCACCTGCAGCTC

**Table 2:** Hematologic parameters and red cell indices in aging wild-type and Nrf2^−/−^ mice.

	Wild-Type Mice		
	**4-Month-Old** **Mice (*n* = 6)**	**8-Month-Old** **Mice (*n* = 6)**	**12-Month-Old** **Mice (*n* = 6)**
**Hct (%)**	46.1 ± 1.4	45.9 ± 0.7	44.8 ± 0.2
**Hb (g/dL)**	14.8 ± 0.5	15.0 ± 0.1	14.3 ± 0.4
**MCV (fl)**	51.3 ± 0.2	51.0 ± 0.1	52.2 ± 0.3
**MCH (pg)**	15.9 ± 0.7	16.5 ± 0.3	15.6 ± 0.2
**RDW (%)**	12.4 ± 0.08	13.5 ± 0.1	12.7 ± 0.3
**Retics (10^3^ cells/uL)**	450 ± 22	431 ± 51	248 ± 24 °
**MCVr (fl)**	54.9 ± 2	56.7 ± 3	59.9 ± 1.8 °
	**Nrf2^−/−^** **Mice**		
	**4-Month-Old Mice** **(*n* = 6)**	**8-Month-Old Mice** **(*n* = 6)**	**12-MonthsOld Mice** **(*n* = 6)**
**Hct (%)**	44.3 ± 0.8	41.8 ± 1.1 °*	33.6 ± 3 °*
**Hb (g/dL)**	13.2 ± 0.5	12.0 ± 0.2 °*	11.0 ± 0.5 °*
**MCV (fl)**	51.8 ± 1.5	50.0 ± 2.0	57.2 ± 1.3 °*
**MCH (pg)**	16.7 ± 1.1	16.0 ± 0.3	16.1 ± 0.4
**RDW (%)**	13.9 ± 0.55	13.2 ± 0.4	14.1 ± 0.4 °*
**Retics (10^3^ cells/uL)**	380 ± 20 *	190 ± 59 °*	180 ± 12 °*
**MCVr (fl)**	61.2 ± 1.3 *	61.0 ± 1.4 *	65.0 ± 0.2 °*

Hct: hematocrit; Hb: hemoglobin; MCV: mean corpuscular volume; MCH: mean corpuscular hemoglobin; RDW: red cell distribution width; Retics: reticulocytes; * *p* < 0.05 compared to wild-type mice; ° *p* < 0.05 compared to 4-month-old mice.

**Table 3:** Effects of ATS-PLGA nanoparticles on hematological parameters and red cell indices in wild-type and Nrf2^−/−^ mice.

	Vehicle Wild-Type Mice (n = 6)	ATS-NP Wild-Type Mice (n = 6)	Vehicle Nrf2^−/−^ Mice (n = 6)	ATS-NP Nrf2^−/−^ Mice (n = 5)
**Hct (%)**	44.8 ± 0.2	45.9 ± 0.7	33.6 ± 3 *	43.5 ± 0.9 *^
**Hb (g/dL)**	14.3 ± 0.4	15.0 ± 0.1	11.0 ± 0.5 *	14.0 ± 0.4 ^
**MCV (fl)**	52.2 ± 0.3	51.0 ± 0.1	57.2 ± 1.3 *	53.5 ± 1.1 ^
**MCH (pg)**	15.6 ± 0.2	16.5 ± 0.3	16.1 ± 0.4	16.3 ± 0.2
**RDW (%)**	12.7 ± 0.3	13.5 ± 0.1	14.1 ± 0.4 *	12.6 ± 0.4 ^
**Retics (10^3^ cells/uL)**	248 ± 24	431 ± 51 ^	180 ± 12 *	355 ± 65 ^
**MCVr (fl)**	59.9 ± 1.8	56.1 ± 2.0	65.0 ± 0.2 *	62.0 ± 1.6 ^

Hct: hematocrit; Hb: hemoglobin; MCV: mean corpuscular volume; MCH: mean corpuscular hemoglobin; RDW: red cell distribution width; Retics: reticulocytes; * *p* < 0.05 compared to wild-type mice; ^ *p* < 0.05 compared to vehicle-treated animals.

## Data Availability

Detailed materials and methods are reported in Supplemental materials. All the data and protocols are stored in the Nas Synology DS216se Hard Disk, located at the University of Verona, 37134 Verona, Italy and will be available on request.

## Supplementary Materials

The following supporting information can be downloaded at https://www.mdpi.com/article/10.3390/antiox13040454/s1: Supplementary Methods; Figure S1. Expression of Nrf2 dependent antioxidant systems in red cell cytosol fraction and amounts of erythrocytes positive for naturally occurring antibody in young and old wild-type and Nrf2^−/−^ mice; Figure S2. Profile or erythroblast subpopulations and expression of Nrf2-dependent antioxidant systems in sorted erythroblasts from wild-type and Nrf2^−/−^ mice; Figure S3. Brightfield images, immunoblot analysis of peroxiredoxin-2 (Prdx2) nuclear translocation in sorted polychromatic and orthochromatic erythroblasts and *Erfe* gene expression in sorted erythroblasts from wild-type and Nrf2^−/−^ mice; Figure S4. Effects of doxorubicin (Doxo) on hematologic parameters and erythropoiesis in wild-type and Nrf2^−/−^ mice; Figure S5. Localization of HSP70, p62 gene expression and control brightfield in sorted erythroblasts from wild-type and Nrf2^−/−^ mice; Figure S6. Characterization of ATS-NPs and calibration curve. Figure S7. Effects of ATS-NPs on Nrf2^−/−^ mouse erythroblast subpopulations, red cell membrane oxidation, membrane association of HSP70 and 90 and macrophage profile; Table S1. Primers used in the present study. References [11,59,60,61] are cited in the supplementary materials.
