# Supplementary material for: Nrf2 Plays a Key Role in Erythropoiesis during Aging
Source: Antioxidants (Basel). 2024 Apr 12;13(4):454. doi: 10.3390/antiox13040454 (PMC11047311; doi:10.3390/antiox13040454)
Supplement: Supplementary file 1 [file antioxidants-13-00454-s001.zip › antioxidants-2942434-supplementary.pdf]

Supplementary Materials

1. Supplementary Methods
2. Supplementary Figures

1. Supplementary Methods

1.1 Drugs and Chemicals

NaCl, Na_3VO_4, TRIS, Tween 20, EDTA, choline, $MgCl_2$, MOPS, Na_2HPO_4, KH_2PO_4, NaF, bicine, β-mercaptoethanol, benzamidine, glycine, glycerol, potassium cyanide, bromphenol blue, sodium dodecil sulphate (SDS), hydrocortisone, albumin from bovine serum (BSA), May-Grunwald-Giemsa's Azur-Eosin-Methylene Blue solution, Astaxanthin, Poly (D,L-lactide-co-glycolide) (PLGA) were obtained by Sigma/Aldrich (7647-14-5, 13721-39-6, 77-86-1, 9005-64-5, 4005-OP, C2004, M1028, M1254, 567550, P5379, S6776, 391336-M, 444203, 12072, G7126, G5516, 207810, B3269, 7990-OP, 1319002, A7030, W0625, SML0982, 805106 Sigma/Aldrich St Louis, MO, USA); dithiotreithol (DTT), was from Fluka (15895458 Buchs, Switzerland); protease inhibitor cocktail tablets were from Roche (11697498001 Basel, Switzerland); Prestained protein ladder, Triton X-100 and Temed were purchased from Sigma/Aldrich (94964, 93443, T9281 St Louis, MO, USA); 40% Acrylamide/Bis Solution, 37.5:1 was from BIO-RAD (161-0154 California, USA); Luminata Forte and Luminata classico Western Hrp solutions were from Mercks Group (ELLUF0100, WBLUC Armstad, Germany); Annexin V Binding Buffer was from eBioscience (BMS500BB San Diego, USA); Dulbecco's Phosphate Buffered Saline (DPBS) was from Lonza (51226 Belgium). Alpha-MEM, L-glutamine and Fetal Cow Serum (FCS) were from ThermoFisher (12571063, A2916801, A5669801 Massachusetts, USA); Penicillin-Streptamicin and Amphotericin were from Euroclone (15140122, 15290026 Milan, Italy); MethoCult™ M3234 was from StemCell Techologies (Milan, Italy).

1.2 Immunomicroscopic analysis of sorted erythroid precursors

Total erythroblasts (CD44+Ter119+FSChigh) were sorted from WT and Nrf2$^{-/-}$ mouse bone marrow using a FACS Aria-IIITM cell sorter (Becton Dickinson, San Jose, CA, USA) as previously described [1]. 200,000 sorted cells were cyto-spun onto poly-lysinated glass slides and fixed with 4% paraformaldehyde added of sucrose 4% in PBS for 20′ at RT. Slides were washed three times with PBS, BSA 1% and permeabilized with PBS, BSA 1%, Triton X-100 0.3% for 10 min at RT. After 3 additional washes with PBS, BSA 1%, unspecific bindings were blocked with PBS BSA 1% for 1h at RT. Cells were then stained with anti-Nrf2 or anti-Rab5 (ab62352, ab109012 Abcam, Cambridge, UK) or anti-Prdx2 (ab109367 Clone1E8, Abcam, Cambridge, UK), O/N at 4°C. After 3 washes with PBS BSA 1%; cells were stained with secondary anti-rabbit or anti-mouse IgG antibody Alexa Fluor 633, Alexa Fluor 488 or Alexa Fluor 546 conjugated (A-21050, A10680, A-11010 Thermo Fisher Scientific, dilution 1/100) for 1h at RT. Nuclei were stained with DAPI (62248 Thermo Fisher Scientific, dilution 1/2000) in PBS BSA 1% for 10 min at RT. After 3 washes, slides were mounted and blocked with nail polish. Images were captured using a LEICA TCS-SP5 Inverted Confocal Microscope equipped with a high NA oil immersion 63x objective. Images were analysed using the ImageJ software as previously described [1] for nuclear/Cytosol distribution and following the particle analysis instructions for large cluster counting (https://imagej.net/imaging/particle-analysis).

1.3 Chip analysis

Sorted erythroblasts were crosslinked and chromatin was extracted and sonicated. Chromatin was incubated overnight with the following antibodies: IgG (X0720 Vector Laboratories, 1:50), anti-Prdx2 (ab109367 AbCam, clone 1E8, 1:50). Dynabeads Protein G were blocked overnight with 1mg/ml Sonicated Salmon Sperm (15632011 Thermo Fisher Scientific) and 1mg/ml Bovine Serum Albumin (AM2616 Thermo Fisher Scientific). 30◎l Dynabeads Protein G (50% slurry) were used for each IP. Immunprecipitated chromatin was purified with DNeasy Blood & Tissue Kit (69504 Qiagen) as previously described [2].

Quantitative PCR was used to assess for DNA enrichment. Immunoprecipitated chromatin was normalized to input chromatin (GAPDH gene). Histograms show immunoprecipitated chromatin relative to control IgG.

The following primer sequences were used:

Site 1 (*Erfe*) Fw: 5′- GGTTCCCCAGCTTGAAAAGG-3′;

Site 1 (*Erfe*) Rev: 5′- CCGGGTCTCTTCTTTTCTCATG-3′;

Site 2 (*Erfe*) Fw: 5′- GGTTCCCCAGCTTGAAAAGG -3′;

Site 2 (*Erfe*) Rev: 5′- CCGGGTCTCTTCTTTTCTCATG -3′;
Site 1 (*Ho-1*) Fw: 5′- GTCAGCAAACCAAGCAGAACTG-3′;
Site 1 (*Ho-1*) Rev: 5′- CAGGGATTGACCCACACTACCT-3′;
Site 2 (*Ho-1*) Fw: 5′- TGGAGAGATGGCCTGTGGTT-3′;
Site 2 (*Ho-1*) Rev: 5′- GAACTGGGAATTAAGGTGGTAGTGA-3′;
NQO1 Fw: 5′- GGTATGCCCTGTATGACTCGCTA-3′;
NQO1 Rev: 5′- CTGACTGGCCAGGAGAAAAGG-3′;
GAPDH Fw: 5′-CTTACCTGTGCTCCCACTCCTGATTT-3′;
GAPDH Rev: 5′-CAGCTGCACCCTTTAGGGAGAAAA-3′.
Quantitative Real-Time PCR has been performed using PowerUP SYBR Green Master Mix (A25742 Applied Biosystems) following manufacturer's instruction.

1.4 Mass spectrometric analysis of plasma and organs ATS-NPs distribution

1.4.1 Extraction of astaxanthin from serum

An aliquot of serum (30 µl) was diluted with a double volume of ethanol to precipitate the proteins, whereas six volumes of hexane (180 µl) were added for the extraction of astaxanthin twice for a double extraction [3]. After every addition of hexane, the upper layer was collected by centrifuging at 12000 rpm for 10 min at 4°C, and the combined hexane layers were dried by a speed-vacuum concentrator. The dried hexane extract was redissolved in 30 µL of methanol acidified by 0.1% formic acid for further LC-MS/MS analysis.

1.4.2 Extraction of astaxanthin from tissues

Each tissue, i.e. liver (L), spleen (SP), and kidney (K), was weighted (roughly 30 mg) according to the material availability, suspended in 200 µl of lysis buffer (8 M urea, 75 mM NaCl in 100 mM NH4HCO3, 10 mM NaF at pH 7.8) and homogenized by T 10 basic Ultra-Turrax (IKA Dispersers). A double volume of ethanol and six volumes of hexane were used as described for the astaxanthin extraction from sera. The combined hexane layers were dried and resuspended in 50 µL of methanol acidified by 0.1% formic acid for the LC-MS/MS analysis.

1.4.3 LC-MS/MS instrumentation and conditions

The supernatant (2 µL) was analysed by using an Agilent 6420 Triple Quadrupole LC-MS/MS system with an HPLC 1100 series binary pump (Agilent, Waldbronn, Germany). The mixture was separated on a Kinetex 5µm C18 100A HPLC Column, by the mobile phase A (H_20 containing 5 mM ammonium formate and 0.1% formic acid) and B (methanol in 0.1% formic acid). The flow rate was at 0.3 mL/min. The elution gradient was from 30% to 95% B in 9 min. Tandem mass spectrometry was performed by using a turbo ion spray source operated in positive ion mode, and the MRM ion mode was used for the selected analytes [4]. A list of MRM parameters set up for astaxanthin analysis including precursor ion, product ions collision energy (CE) and dwell time is reported in Table S1.

Table S1. List of MRM parameters set up for astaxanthin analysis

Analyte	Precursor ion m/z	Daughter ion m/	Dwell time	Collision Energy (CE)
**Astaxanthin**	597.0	579.1	25	8
	597.0	561.5	25	15
	597.0	505.4	25	18
	597.0	473.2	25	20
	597.0	379.2	25	20
	597.0	173.3	25	30
	597.0	147.0	25	40

1.5 References

1. Matte, A.; De Falco, L.; Iolascon, A.; Mohandas, N.; An, X.; Siciliano, A.; Leboeuf, C.; Janin, A.; Bruno, M.; Choi, S.Y.; et al. The Interplay Between Peroxiredoxin-2 and Nuclear Factor-Erythroid 2 Is Important in Limiting Oxidative Mediated Dysfunction in beta-Thalassemic Erythropoiesis. *Antioxid Redox Signal* **2015**, *23*, 1284-1297, doi:10.1089/ars.2014.6237.
2. Santoro, R.; Zanotto, M.; Carbone, C.; Piro, G.; Tortora, G.; Melisi, D. MEKK3 Sustains EMT and Stemness in Pancreatic Cancer by Regulating YAP and TAZ Transcriptional Activity. *Anticancer Res* **2018**, *38*, 1937-1946, doi:10.21873/anticanres.12431.
3. Colman-Martinez, M.; Martinez-Huelamo, M.; Miralles, E.; Estruch, R.; Lamuela-Raventos, R.M. A New Method to Simultaneously Quantify the Antioxidants: Carotenes, Xanthophylls, and Vitamin A in Human Plasma. *Oxid Med Cell Longev* **2015**, *2015*, 9268531, doi:10.1155/2016/9268531.
4. Dattilo, M.; Fontanarosa, C.; Spinelli, M.; Bini, V.; Amoresano, A. Modulation of Human Hydrogen Sulfide Metabolism by Micronutrients, Preliminary Data. *Nutr Metab Insights* **2022**, *15*, 11786388211065372, doi:10.1177/11786388211065372.

2. Supplementary Figures

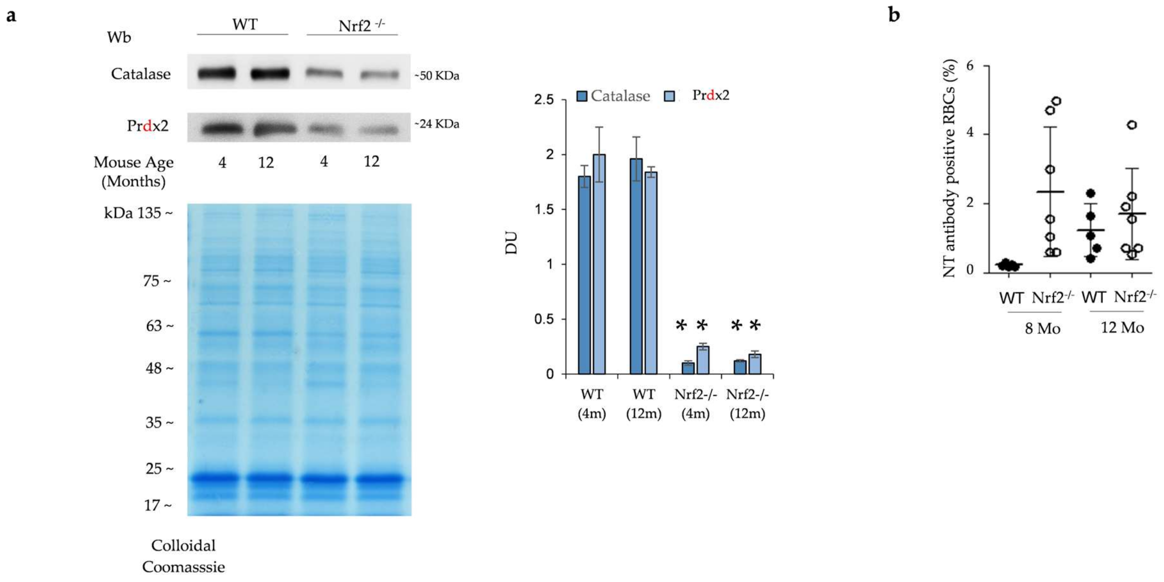

Figure S1. Expression of Nrf2 dependent antioxidant systems in red cell cytosol fraction and amounts of erythrocytes positive for natural occurring antibody in young and old wild-type and Nrf2$^{-/-}$ mice. (a) Western blot (Wb) analysis using specific antibodies against catalase and Prdx2 of the cytosolic fraction of red cells from WT and Nrf2$^{-/-}$ mice at 4 – 12- months of age. One representative gel of other four is shown. Colloidal Coomassie staining was used as loading control. Densitometric analysis of immunoblots is shown in the right panel. Data are presented as means ±SD (n=4); * p<0.05 compared to WT mice; (**b**) Flow cytometric analysis of the red cells carrying natural (NT) antibody on membrane surface from WT and Nrf2$^{-/-}$ mice at 8- and 12-months of age. Data are presented as single dots (n= 5-7).

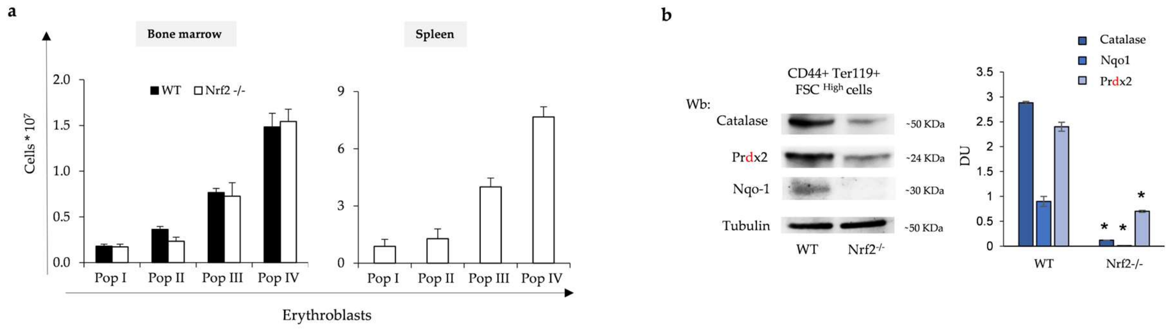

Figure S2. Profile or erythroblasts subpopulations and expression of Nrf2-dependent antioxidant systems in sorted erythroblasts from wild-type and Nrf2$^{-/-}$ mice. (**a**) Flow-cytometric analysis, combining CD44-Ter119 and cell size marker strategy (CD44+/Ter119+/FSC), of the erythropoietic subpopulation (Pop I corresponding to Pro-Erythroblasts, Pop II to Basophilic-Erythroblasts, Pop III to Polychromatic-Erythroblasts, Pop IV to Orthochromatic-Erythroblasts) in the bone marrow and spleen from wild-type (WT) and Nrf2$^{-/-}$ mice. Data are presented as means ±SD (n=4). (**b**) Wb analysis with specific anti catalase, Prdx2, Nqo-1 in sorted erythroid precursors from bone marrow of WT and Nrf2$^{-/-}$ mice. Tubulin was used as protein loading control. Densitometric analysis of immunoblots is shown in the right panel. Data are presented as means ±SD (n=4); * p<0.05 compared to WT mice.

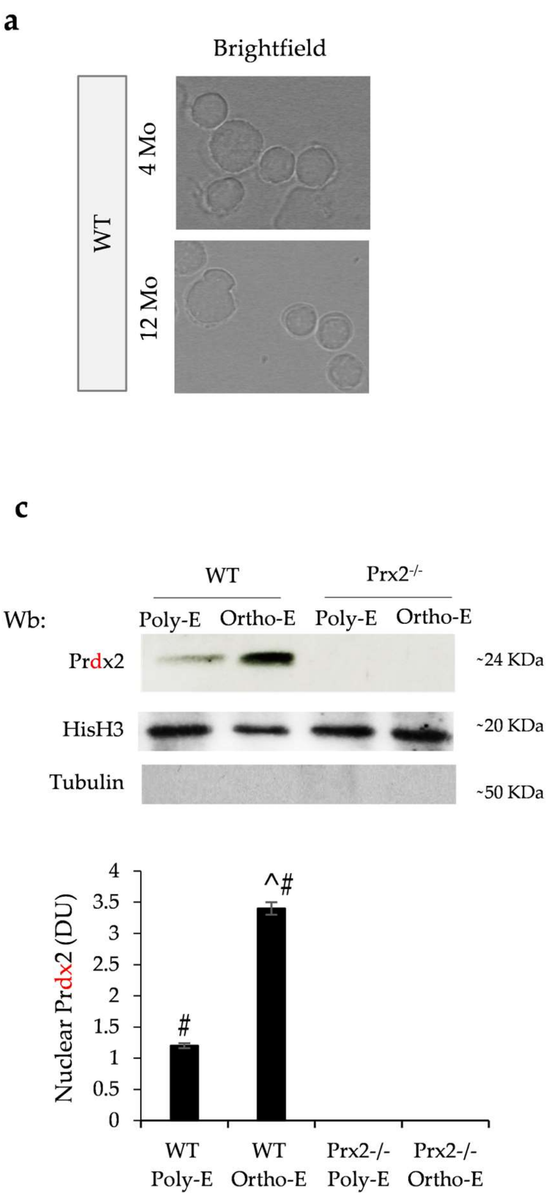

Figure S3. Brightfield images, immunoblot analysis of peroxiredoxin-2 (Prdx2) nuclear translocation in sorted polychromatic and orthochromatic erythroblasts, nuclear localization of Prdx2 in sorted erythroblasts from wild-type and Prdx2$^{-/-}$ mice, and *Erfe* gene expression in sorted erythroblasts from wild-type and Nrf2$^{-/-}$ mice. (**a**) Brightfield of the Nrf2 immunostained fluorescence images shown in Figure 3a (**b**) Brightfield of the Prdx2 immunostained fluorescence images shown in Figure 3d (**c**) Wb analysis using specific anti Prdx2 of the nuclear fraction of the sorted polychromatic (Poly) or orthochromatic (Ortho) erythroblasts (E) using the CD44+/Ter119+/FSC gating strategy from WT and Nrf2$^{-/-}$ mice. HisH3 was used as loading control. Densitometric analysis of immunoblots is shown in the lower panel. # $p<0.05$ compared to Prdx2$^{-/-}$ Poly and Ortho-E; ^ $p<0.05$ compared to WT Poly-E; (**d**) mRNA expression of *Erfe* by qRT-PCR on the erythroblasts from WT and Nrf2$^{-/-}$ mice (fold on WT). Experiments were performed in triplicate. $^{*}p < 0.01$, Nrf2$^{-/-}$ vs WT mice. P value was calculated by t-test.

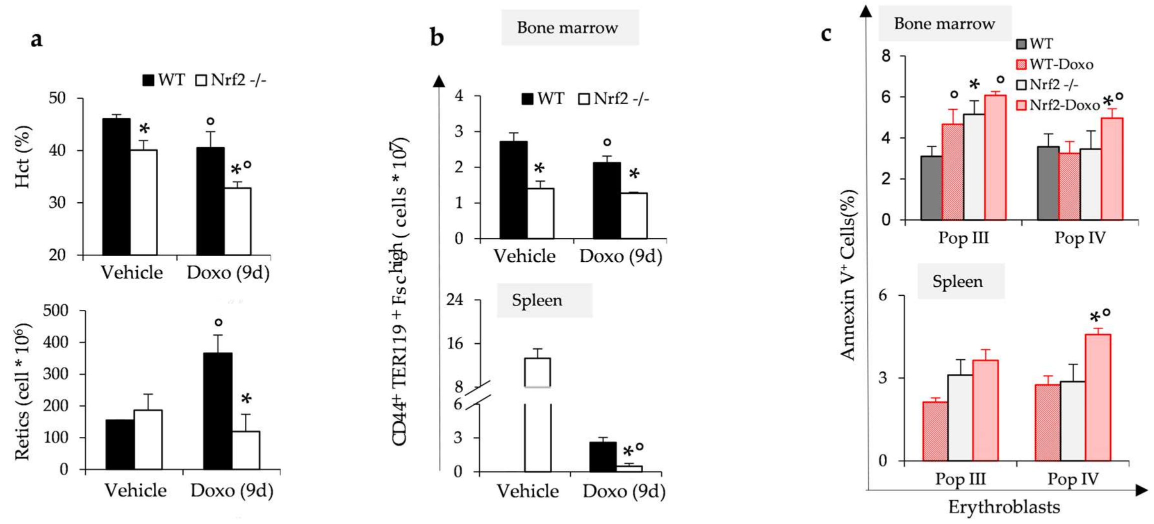

Figure S4. Effects of doxorubicin (Doxo) on hematologic parameters and erythropoiesis in wild-type and Nrf2$^{-/-}$ mice. (**a**) Hematocrit (Hct) and Reticulocyte count in WT and Nrf2$^{-/-}$ mice 9 days after Vehicle or Doxorubicin (0.25 mg/Kg body weight) administration. Data are presented as means±SD (n=6). *p<0.05 compared to WT; °p<0.05 compared to vehicle. (**b**) Flow-cytometric analysis, combining CD44-Ter119 and cell size marker strategy (CD44+/Ter119+/FSChigh), of the erythropoietic activity in the bone marrow and spleen from WT and Nrf2$^{-/-}$ mice treated as in a. Data are presented as means±SD (n=6). *p<0.05 compared to WT; °p<0.05 compared to Vehicle. (**c**) Annexin V+ cells in population III (corresponding to polychromatic erythroblasts) and population IV (corresponding to orthochromatic erythroblasts) from either spleen or bone marrow of WT and Nrf2-/-mice respectively ad day 9 after Doxo administration. Data are presented as means±SD (n=6) *p<0.05 compared to WT; °p<0.05 compared to vehicle.

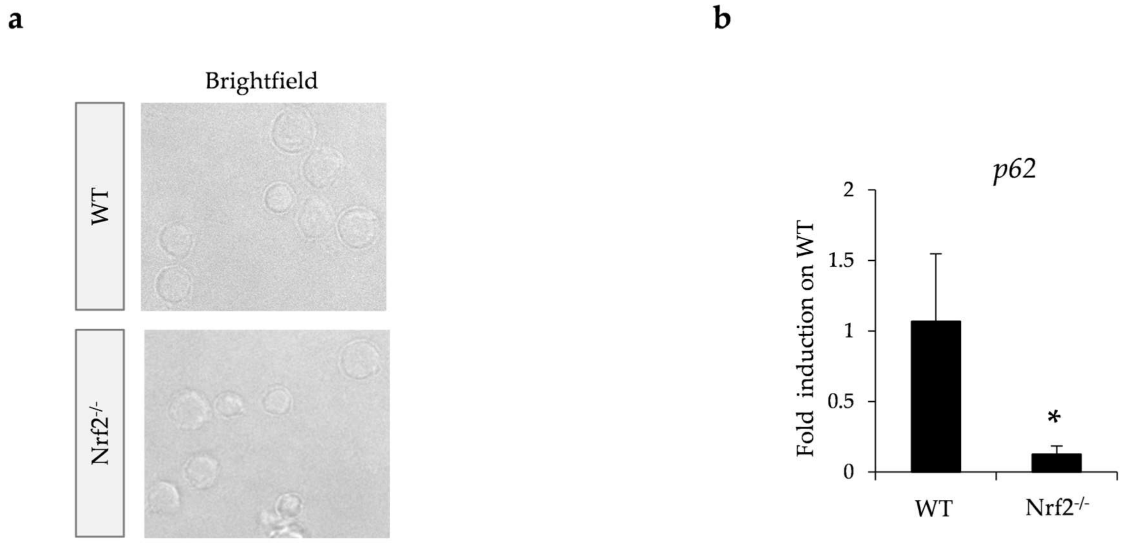

Figure S5. control brightfield and p62 gene expression in sorted erythroblasts from wild-type and Nrf2$^{-/-}$ mice. (**a**) Brightfield of the Rab5 immunostained fluorescence images shown in Figure 4e. (**b**) mRNA expression of *P62* by qRT-PCR on the erythroblasts from WT and Nrf2$^{-/-}$ mice (fold on WT, n=4). Experiments were performed in triplicate. *p < 0.01, Nrf2$^{-/-}$ *vs* WT mice. P value was calculated by t-test.

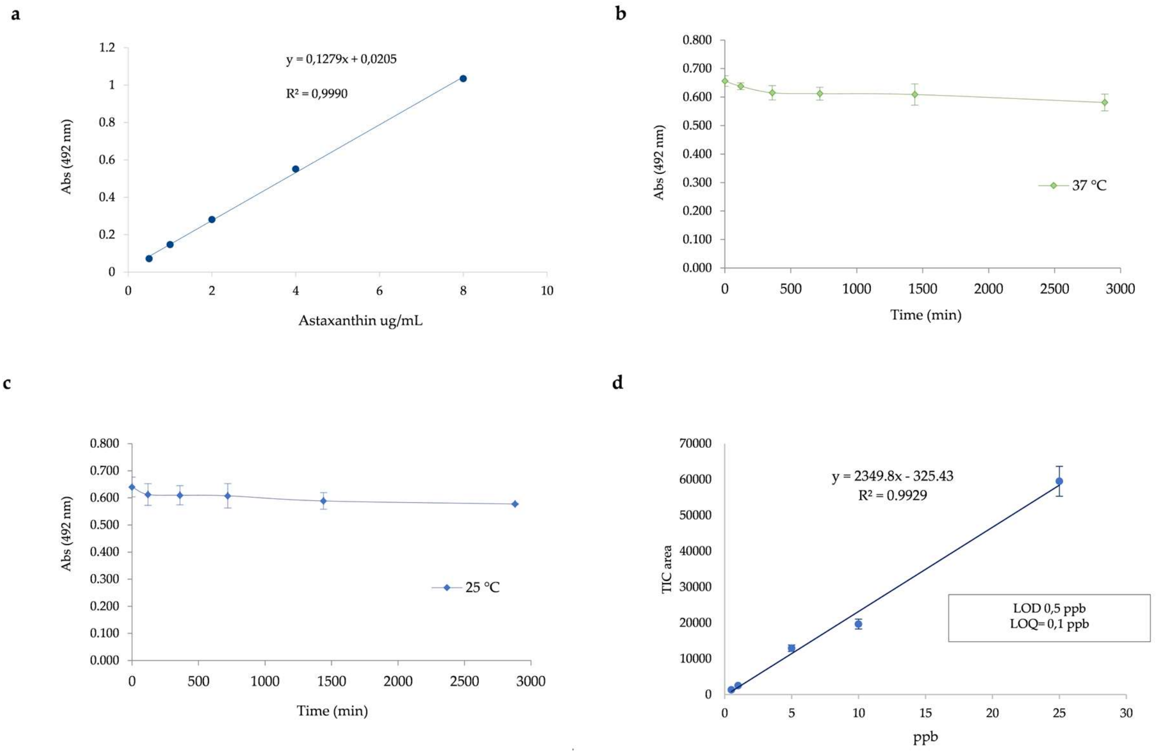

Figure S6. Characterization of ATS-NPs and calibration curve. (**a**) Calibration curve for quantification of astaxanthin (ATS) in PLGA NPs obtained dissolving different amounts of carotenoid in 1 ml of dimethyl sulfoxide (DMSO) and reading the absorbance of each solution at 492 nm. (**b-c**) Astaxanthin stability in PLGA NPs measured as the decreasing of the absorbance at 492 nm over the time respectively at 37 °C as well at 25 °C. Measurement were performed after dissolving the nanoparticles in DMSO and are means of three independent measures on three replica samples. (**d**) Extracted ion chromatogram for astaxanthin illustrating the calibration curve obtained for astaxanthin.

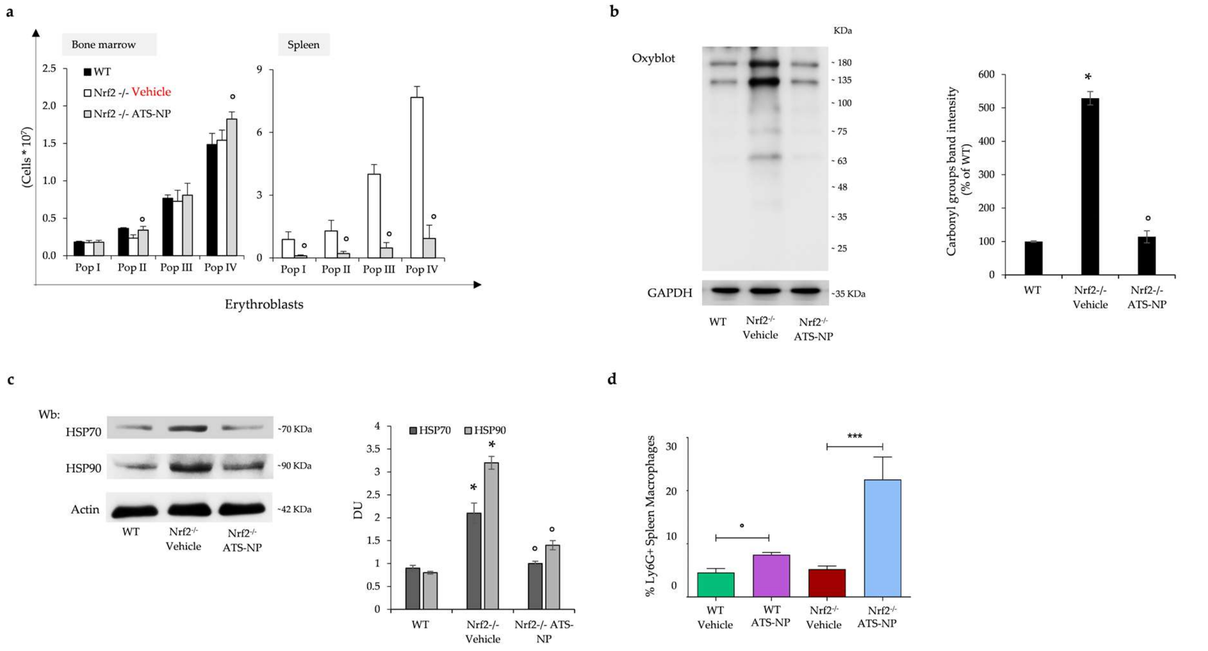

Figure S7. Effects of ATS-NPs on Nrf2$^{-/-}$ mouse erythroblasts subpopulations, red cell membrane oxidation, membrane association of HSP70, 90 and macrophage profile. (**a**) Flow-cytometric analysis, combining CD44-Ter119 and cell size marker strategy (CD44+/Ter119+/FSC), of the erythropoietic subpopulation (Pop I corresponding to Pro-Erythroblasts, Pop II to Basophilic-Erythroblasts, Pop III to Polychromatic-Erythroblasts, Pop IV to Orthochromatic-Erythroblasts) in the bone marrow and spleen from wild-type (WT) and Nrf2$^{-/-}$ mice treated with vehicle or ATS-NPs (2 mg/Kg every two days for four weeks). Data are presented as means ±SD (n=4); ° p<0.05 compared to vehicle treated Nrf2$^{-/-}$ mice. (**b**) OxyBlot analysis of the ghost fraction of red cells from WT and Nrf2$^{-/-}$ mice as in a. GAPDH was used as loading control. Densitometric analysis is shown in the right panel. Data are presented as means ±SD (n=4); * p<0.05 compared to WT mice, °p<0.05 compared to vehicle treated mice. (**c**) Wb analysis using specific antibodies against HSP70 and HSP90 of the ghost fraction of red cells from WT and Nrf2$^{-/-}$ mice treated as in a. One representative gel of other four is shown (upper panel). Actin is used as protein loading control. Densitometric analysis of immunoblots is shown in the right panel. Data are presented as means ±SD (n=4); * p<0.05 compared to WT mice, ° p<0.05 compared to vehicle treated mice. (**d**) Flow cytometry analysis of phagocytosis of Ly6G^{+} cells by spleen macrophages. Data are mean ± SD (n = 6); ***, p<0.001; ° p<0.05 by 1-way ANOVA.
